# SEDEX—Self-Emulsifying Delivery Via Hot Melt Extrusion: A Continuous Pilot-Scale Feasibility Study

**DOI:** 10.3390/pharmaceutics14122617

**Published:** 2022-11-27

**Authors:** Ožbej Zupančič, Aygün Doğan, Josip Matić, Varun Kushwah, Carolina Alva, Martin Spoerk, Amrit Paudel

**Affiliations:** 1 Research Center Pharmaceutical Engineering GmbH (RCPE), Inffeldgasse 13, 8010 Graz, Austria; 2 Institute of Process and Particle Engineering, Graz University of Technology, Inffeldgasse 13/3, 8010 Graz, Austria

**Keywords:** self-emulsifying drug delivery systems (SEDDSs), hot melt extrusion (HME), ZSK18, pilot scale production, solid SEDDSs, SEDDSs characterization

## Abstract

The aim of this study was to develop a continuous pilot-scale solidification and characterization of self-emulsifying drug delivery systems (SEDDSs) via hot melt extrusion (HME) using Soluplus^®^ and Kollidon^®^ VA-64. First, an oil-binding capacity study was performed to estimate the maximal amount of SEDDSs that the polymers could bind. Then, HME was conducted using a Coperion 18 mm ZSK18 pilot plant-scale extruder with split-feeding of polymer and SEDDS in 10, 20, and 30% *w*/*w* SEDDSs was conducted. The prepared extrudates were characterized depending on appearance, differential scanning calorimetry, wide-angle X-ray scattering, emulsification time, droplet size, polydispersity index, and cloud point. The oil-binding studies showed that the polymers were able to bind up to 50% *w/w* of liquid SEDDSs. The polymers were processed via HME in a temperature range between 110 and 160 °C, where a plasticizing effect of the SEDDSs was observed. The extrudates were found to be stable in the amorphous state and self-emulsified in demineralized water at 37 °C with mean droplet sizes between 50 and 300 nm. A cloud point and phase inversion were evident in the Soluplus^®^ samples. In conclusion, processing SEDDSs with HME could be considered a promising alternative to the established solidification techniques as well as classic amorphous solid dispersions for drug delivery.

## 1. Introduction

Oral dosage forms represent about 80% of the pharmaceutical market due to their convenience, cost-effectiveness, non-sterile manufacturing, self-administration, and high solid dosage in terms of manufacturing technology readiness level [1,2]. Among them, oral solids pose additional industrial, economical, drug product quality, and formulation benefits. For instance, solid dosage forms offer numerous downstream possibilities to form, e.g., tablets, capsules, powders, dry emulsions, pellets, and sachets [3,4]. In addition, solid dosage forms are less bulky, enable exact dosing with each dosage unit as well as show flexibility in terms of scalability, equipment, process analytical technology (PAT) and have a long track record in pharmaceutical good manufacturing practice (GMP) [5]. Hence, it is of great interest to convert liquid formulations such as self-emulsifying drug delivery systems (SEDDSs) into solid forms.

SEDDSs are isotropic preconcentrates of lipids, surfactants, and co-solvents in various ratios. Upon contact with aqueous media, SEDDSs spontaneously emulsify, resulting in an effective solubilization of the incorporated drug in the lipid core by rapid and homogeneous dispersion in the medium [5,6,7]. SEDDSs are considered as one of the most promising oral delivery systems due to their ability to mimic endogenous lipid metabolism, creating a local amphiphilic microenvironment and thus improving drug solubilization and permeation [3,4]. 

Liquid SEDDSs have already successfully been converted into solid form via spray drying, freeze drying, or adsorption on various meso- and micro-porous inorganic, colloidal, and polymeric solid carriers [8,9,10,11]. In addition, solidification techniques utilizing an extruder were performed, where twin-screw wet/melt granulation with a subsequent spheronization step was most common [12,13,14]. In contrast, hot melt extrusion (HME) remains an overlooked solidification technique in the SEDDSs field. HME is a continuous manufacturing process used to compound, melt, and mix powder or liquid raw materials into an intermediate or final solid dosage form. The individual components are fed into the extruder via one or multiple powder or liquid feeders and processed along the co-rotating double screws. The HME process is flexible and allows for the screw configuration to be tailored to the specific requirements of the processed formulation [15,16]. In addition, HME offers the possibility of implementing PAT, real-time quality monitoring [15,17,18], and other tools for advanced process control [18,19,20,21]. Recently, in silico process development and scale-up has shown successful cases of reducing the overall process development time and material waste [21,22,23,24]. With this, HME has become a reliable process route for amorphous solid dispersions (ASDs) with relatively simple simulation guided scale-up. The process with tailored screw configuration, multiple feeding, and degassing options enable for novel formulations and solidification processes [5,25,26,27,28].

However, few publications where HME was used to solidify SEDDSs in a pure polymer matrix are available. In one study, HME solidification of carvedilol SEDDSs was performed by pre-adsorbing SEDDSs on the solid matrix composed of microcrystalline cellulose (MCC), colloidal silicon dioxide, talc, and a mixture of hydroxy propyl methylcellulose-acetate/succinate (HPMCAS) and hydroxy propyl cellulose (HPC) [29]. To the best of our knowledge, no published data on preparing solid SEDDSs by HME on a pilot scale exist. Therefore, it was the aim of this study to demonstrate a proof-of-concept solidification of model SEDDSs formulation in a polymer matrix by pilot-scale HME via a continuous split feeding process. The extruder of choice was the 18 mm ZSK18 co-rotating twin screw extruder from Coperion. Soluplus^®^ (SOL) and Kollidon^®^ VA-64 (VA-64) were chosen as typical HME polymers. Prior to HME, the SEDDSs’ sorbing capacity on polymers was evaluated to estimate the optimal polymer to SEDDSs ratio. Afterward, the HME process was performed continuously by varying the SEDDSs–polymer ratio via feeding rates in real-time. The resulting extrudates were characterized in terms of appearance, differential scanning calorimetry (DSC), wide angle X-ray scattering (WAXS), emulsification time, droplet size, polydispersity index (PDI), and cloud point.

## 2. Materials and Methods

### 2.1. Materials

Kollidon^®^ VA-64 (copovidone, VA64), Soluplus^®^ (Poly(vinyl caprolactam-covinylacetate-ethylene glycol) graft polymer, SOL), and Kolliphor^®^ RH40 (PEG-40 Hydrogenated Castor Oil) were received from BASF SE (Ludwigshafen am Rhein, Germany). Plasdone^®^ S-630 (PVPVA64, PL_630) and Plasdone^®^ S-630 Ultra (PVPVA64, PL_630U) were delivered from Ashland Inc. (Düsseldorf, Germany). Capmul^®^ MCM EP (Glycerol Monocaprylocaprate Type I EP) was provided by Abitec Corp (Janesville, WI, USA). Transcutol^®^ (diethylene glycol monoethyl ether) and Labrafac^®^ lipophile WL 1349 (medium chain triglycerides) were received from Gattefossé (Saint-Priest, France).

### 2.2. Liquid SEDDS Preparation and Polymer Solubility

Liquid SEDDS were prepared as described previously [7,30]. First, semi-solid Kolliphor^®^ RH40 was melted in a water bath (50 °C) for 30 min. Then, 30 g of Kolliphor^®^ RH40, 30 g of Capmul^®^ MCM, 30 g of Labrafac^®^ lipophile WL 1349, and 10 g of Trancutol^®^ were added to a 250 mL glass beaker and stirred on the magnetic stirrer Heildorph MR HEI Standard (Schwabach, Germany) at 200 rpm overnight at room temperature. 

The polymer solubility in the SEDDS was tested qualitatively as follows: 1% *w*/*w*, 5% *w*/*w,* and 10% *w*/*w* of dried polymers were mixed with 99%, 95%, or 90% *w*/*w* of SEDDSs in 1.5 mL Eppendorf tube, corresponding to 1%, 5%, and 10% polymer concentrations, respectively. The samples were sealed and stored protected from light for five days at room temperature for polymers to completely dissolve. Then, the samples were mixed using a vortex mixer and centrifuged with Hettich Universal 320 R (Tuttlingen, Germany) to induce phase separation or sedimentation of the undissolved polymer. The solubility was evaluated visually.

### 2.3. Drying of Polymers

Before conducting experiments, all polymers were dried in the drying oven Memmert UNB 500 (Schwabach, Germany) at 50 °C for 48 h or until a loss on drying (LOD) < 2.0% was reached. The moisture content of polymers was determined in triplicate by heating ~4 g of sample to 105 °C until a mass change of less than 1 mg/90 s was observed. The measurements were performed on the halogen moisture analyzer Mettler Toledo LP 16 (Zaventem, Belgium). 

### 2.4. Oil-Binding Capacity

An oil-binding capacity study was performed by a slightly modified method as described previously [31]. In brief, to 1200 mg (60% *w*/*w*) or 1000 mg (50% *w*/*w*) of dried polymer, 800 mg (40% *w*/*w*) or 1000 mg (50% *w*/*w*), respectively, of liquid SEDDS was added dropwise in a 10 mL glass tube to yield the total sample weight of 2000 mg. The glass tube was then heated with a heating gun, namely Makita HG 5030 (Kortenberg, Belgium), for ~10 min or until the sample visually melted into one homogenous phase. The hot melt was rapidly poured into a 1.5 mL Eppendorf tube and let to cool down and solidify at room temperature for 1 h. After sample solidification, the tubes were centrifuged with Hettich Universal 320 R (Tuttlingen, Germany) for 10 min, 20 ˚C, and 10,000 rpm to induce phase separation of potential unbound SEDDSs. After centrifugation, Eppendorf tubes were tuned upside-down for 1 h at room temperature and let the excess SEDDSs drip out. The oil-binding capacity was calculated as the difference between the weights before centrifugation and post-oil dripping. 

### 2.5. Hot Melt Extrusion 

**Figure 1:**
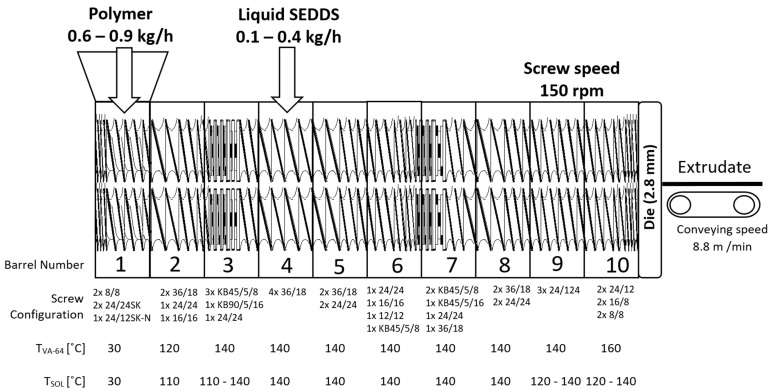
Hot melt extrusion setup with process settings for Kollidon^®^ VA-64 (VA-64) and Soluplus^®^ (SOL) for the preparation of SEDDSs via extrusion (HME–SEDDSs).

A detailed HME process with screw configuration is illustrated in Figure 1. The setup consisted of one K-Tron KT20 feeder equipped with coarse–concave screws (Coperion GmbH, Germany), the ZSK18 co-rotating twin screw extruder with a screw diameter of 18 mm and L/D of 40 equipped with a 2.8 mm die (Coperion GmbH, Stuttgart, Germany), a Geppert conveyor belt (constant conveying speed of 8.8 m/min), and an adapted cooling air tunnel (Dorner GmbH, Jülich, Germany). The conveyor belt was used to keep the exudate in horizontal position and facilitate its solidification after extrusion through the die. This kept the final products in a cylindrical shape and reduced bending or deformations of extrudate to enable flawless potential down-streaming processes. 

The feeding of polymers and liquid SEDDSs was performed separately with a total throughput of 1.0 kg/h. Polymer powders were continuously fed in the first extruder segment, whereas liquid SEDDSs were fed in the fourth. A peristaltic pump, Ismatec MV-CA8 (Zürich, Switzerland), was used to feed liquid SEDDSs with varying speeds between 6 and 18 rpm, corresponding to 0.1–0.3 kg/h. The peristaltic pump was calibrated by generating a calibration curve (R^2^ = 0.9993) to establish the relationship between pump rpm and g/min units. The screw speed of 150 rpm and the screw configuration were kept constant. Prior to the split feeding approach, preliminary co-feeding HME trials on a small-scale 9 mm tabletop extruder Three-Tec TTZE9 (Seon, Switzerland) as well as on a Coperion ZSK18 first segment was attempted and failed due to recurring clogging of the powder intake.

The screw configuration was designed for split feeding process requirements. The powder intake and densification zones were facilitated with a combination of special Schubkanten conveying elements (24/24 SK and 24/12 SK-N) with a pitch of 24 mm and an array of normal conveying elements with a pitch reduction from 36 mm to 16 mm before the next zone. The pitch reduction was introduced to densify the powder and increase the pressure build-up capacity of the screws before the powder melting zone. The powder melting was facilitated in a dedicated zone with the use of 45° and 90° kneading elements with different kneading block thicknesses. Such kneading block assembly provided additional energy input which was intended to fully melt the powder to form a physical barrier to prevent SEDDSs from flowing back in the opposite direction to the segments 1–3. 

The above mentioned assembly of conveying elements also provided a large free volume for the side feeding of liquid SEDDSs into the polymer melt. After feeding, a dedicated mixing zone was assembled from a series of 45° kneading elements with different kneading block thicknesses. The goal of this mixing zone was to evenly incorporate SEDDSs into the polymeric matrix. The melt discharge at the die was facilitated with appropriate combination of conveying elements with different pitches and with a pitch reduction toward the die section. The extruded solidified SEDDSs (HME–SEDDSs) were collected in sealed plastic bags, which were additionally stored in aluminum bags, protected from moisture and light until further analysis. 

### 2.6. HME–SEDDSs Characterization

#### 2.6.1. Polarized Optical Microscopy (POM)

POM analysis was performed on HME–SEDDSs samples using the Olympus-BX 60 microscope (Tokyo, Japan), equipped with a JVC TK-C1381 (Tokyo, Japan) color video camera. Briefly, each sample was placed on a glass slide in the hot stage and observed under the microscope using cross-polarized light at room temperature. Captured images were analyzed using the Olympus Microimage software version 4.0 (Tokyo, Japan).

#### 2.6.2. Differential Scanning Calorimetry (DSC) 

The thermal analysis of the raw materials and HME–SEDDSs was performed on the Netzsch DSC 204F1 Phoenix (Tirschenreuth, Germany) with an auto-sampler. Briefly, 5–10 mg of the samples were enclosed in the pierced 40 µl aluminum pans. The samples were analyzed using the modulated mDSC method, i.e., heated from 0 °C to 150 °C at the rate of 5 °C/min, with periods of 40 s and an amplitude of 0.531. The DSC data were analyzed using the Netzsch Proteus software (Tirschenreuth, Germany).

#### 2.6.3. Wide-Angle X-ray Scattering (WAXS)

WAXS measurements of the HME–SEDDSs were performed using previously described methods [32,33,34]. Briefly, the samples were measured at room temperature using Hecus S3-MICRO system (Bruker) equipped with two linear position sensitive detectors (2Hecus PSD-50, 54 μm/channel). Samples were placed inside X-ray capillaries of 2 mm in diameter and rotated during exposure at ∼0.2 Hz in a temperature-controlled cuvette (TCCS and SpinCap, Hecus). Measurements of the capillaries filled with the samples were performed at an exposure time of 1200 s. The background of the empty capillary was subtracted from the actual sample measurement and the intensities were normalized using the peak area of the primary beam, i.e., scattering mass measured with a Tungsten filter.

#### 2.6.4. Emulsification Time

Emulsification time of HME–SEDDSs was performed similarly as described in [35]. Briefly, HME–SEDDSs were manually micronized using a mortar and sieved through a 1.00 mm sieve. Then, 1.0 g of sieved powder was added into 250 mL of deionized water at 37 °C and the emulsification time was visually observed. The mean emulsification time ± SD was calculated (*n* = 3). 

#### 2.6.5. Droplet Size and Polydispersity Index (PDI)

Samples for droplet size and PDI determination were prepared as follows: 100 mg (2% *w/v*) or 20 mg (0.4% *w/v*) of HME–SEDDSs were emulsified in deionized water via gentle agitation until uniform microemulsion was observed. Then, 0.5–1.0 mL of the prepared microemulsion was analyzed by Litesizer 500 (Anton Paar, Graz, Austria) in automatic mode, calibration time of 60 s, and using the backscatter measurement angle. The droplet size and temperature relationship were measured by Litesizer 500 as follows: 2.0% *w/v* HME–SEDDSs were gradually heated by 1 °C steps, starting at 25 °C until the final temperature of 37 °C was reached. During each stage, droplet size and polydispersity index (PDI) was determined. The measurements were processed and interpreted by the Kalliope software version 2.22.2 (Anton Paar, Graz, Austria). 

#### 2.6.6. Transmittance and Cloud Point 

The cloud point of 2.0% *w/v* HME–SEDDSs was measured by a temperature gradient, where the sample was gradually heated by 1 °C steps, starting at 25 °C until the final temperature of 37 °C was reached. During each stage, sample transmittance was determined. The measurements were processed and interpreted by Kalliope software version 2.22.2 (Anton Paar, Graz, Austria). 

Alternatively, the cloud point was also measured manually. Here, 2.0% *w/v* of HME–SEDDS samples in 5 mL Eppendorf tube were placed in a water bath at 25 °C. The temperature of the water bath was increased gradually with a heating plate Heildorph MR HEI Standard (Schwabach, Germany) and the temperature where the sample turbidity visually increased (sample shifted from transparent to intensive milky white color) was noted as the cloud point. 

## 3. Results and Discussion

### 3.1. Oil-Binding Capacity 

First, an SEDDS formulation containing 30% Kolliphor^®^ RH40, 30% Capmul^®^ MCM, 30% Labrafac Lipohile^®,^ and 10% Transcutol^®^ was chosen as a model formulation, as seen in Table 1.

The chosen SEDDS formulation was slightly modified compared to previous studies—Kolliphor^®^ RH40 was used in place of Kolliphor^®^ EL as hydrophilic non-ionic emulsifier, Transcutol^®^ was used as co-solvent in place of propylene glycol, and Labrafac Lipohile^®^ was used as typical middle-chain triglyceride raw material. Such SEEDS composition could be considered a robust “gold standard” with rapid self-emulsification properties, microemulsion stability, insensitivity to pH, dissolution medium, dilution, and relatively high solubilization capacity [7,30,36].

Before preparing HME–SEDDSs, the capacity of polymers to bind/adsorb SEDDSs was evaluated. Inappropriate polymer to SEDDSs ratios may lead to formulation instabilities such as phase separation, poor HME process performance, and HME–SEDDSs physical state transition after the process (for instance from solid to semi-solid). Preliminary studies showed that all four chosen polymers formed a solid mass when mixed with SEDDSs, heated, and cooled in a 1:1 ratio. Increasing the SEDDSs beyond 50% *w*/*w* yielded in all cases a viscous, sticky, semi-solid mass, inappropriate for HME—a concrete example being 70% SEDDSs in 30% SOL. Hence, to avoid process instabilities and a semi-solid products impossible to downstream after HME, model HME–SEDDSs formulations with an SEDDSs content of 10–30% *w*/*w* were chosen. 

**Figure 2:**
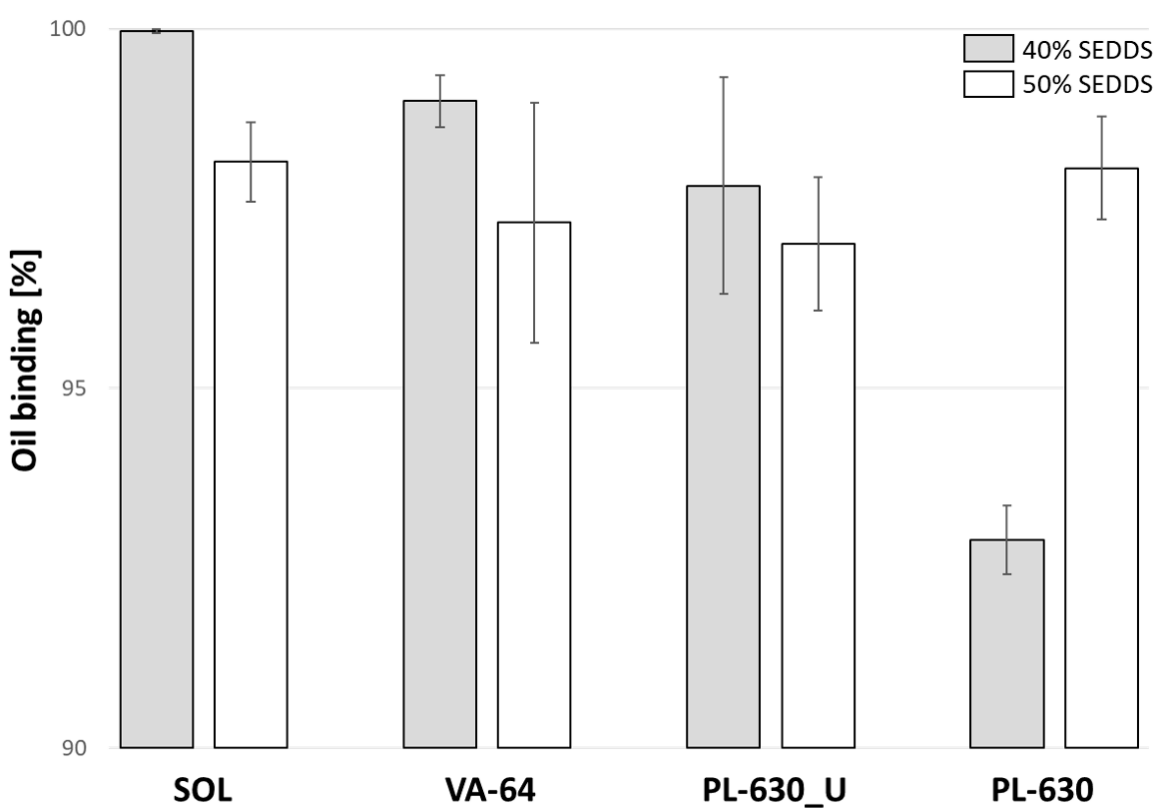
Oil-binding capacity of 40% *w*/*w* SEDDS in 60% *w*/*w* polymer (grey) and 50% *w*/*w* SEDDS in 50% *w*/*w* polymer (white). The polymers used were SOL, VA-64, PL-630_U, and PL-630. Data are shown as mean ± SD (*n* = 3).

Likewise, the oil-binding capacity experiments were set with maximal 40% and 50% *w*/*w* of SEDDSs. The results are shown in Figure 2. All polymers performed similarly and showed >90% oil-binding capacities at both SEDDSs concentrations. However, PL_630 and PL_630U were comparatively more hygroscopic and required a longer time to melt compared with SOL and VA64. In addition, two separate phases were observed in the molten state at PL_630 and PL_630U, indicating poor polymer–SEDDSs miscibility. Therefore, VA64 and SOL were chosen for pilot-scale HME trials. 

### 3.2. Hot Melt Extrusion (HME) 

To begin with, preliminary co-feeding HME trials on both small and pilot-scale extruders failed because immediately upon contact between polymers and SEDDSs, a low-density elastic agglomerate was formed, which floated on top of the screws, blocking the intake port, and was hence unable to be further processed. In this regard, qualitative polymer solubility studies showed that at room temperature, SOL and VA-64 are both soluble in SEDDSs in 1% *w*/*w* and 5% *w*/*w* concentrations. Increasing the polymer concentration to 10% *w*/*w* caused a complete liquefaction of both polymers with two resulting phases, where the sample turned into one turbid phase upon homogenization. The partial polymer solubility in SEDDSs could be an explanation for the agglomerate formation upon contact and failure to process it via co-feeding. It was likely that the polymer was partially dissolved by the SEDDSs and acted as a binder. 

In order to omit the polymer–SEDDSs agglomerate formation, HME was performed on a pilot-scale ZSK18 Coperion, which offers the possibility of feeding the polymer and SEDDSs separately. In this case, the HME process was feasible in 10, 20, and 30% *w*/*w* SEDDSs loading. Surprisingly, 40% *w*/*w* SEDDSs loading yielded a sticky–viscous liquid exiting the die that was not possible to be handled in the extruder downstream. This seems contradictory with the oil-binding capacity studies from above, where polymers were shown to be able to bind up to 50% *w*/*w* of SEDDSs. Nevertheless, in contrast with shorter heating times, lower temperatures, and no shear/mechanical stress in oil-binding studies, the HME process introduced arguably harsher conditions to the polymer–SEDDSs melt, likely being responsible for the non-downstreamable samples at SEDDSs loadings above 30% *w*/*w*. 

Regarding the temperature profiles, increasing the SEDDSs loading resulted in lower process temperatures in certain barrel segments. For instance, the HME temperature profile with VA-64 was constant in all SEDDSs loadings up to 40% *w*/*w*. The temperature profile was set to 30–120–140 °C and kept constant until the final segment, where it was extruded at 160 ˚C. However, processing with SOL was different for 10% SEDDSs compared with 20% and 30% *w*/*w*. For instance, HME–SEDDSs–10%–SOL was processed under a temperature gradient of 30–110–140 °C in the first three segments and kept constant from there on. In contrast, the extrusion of HME–SEDDSs–20%–SOL and HME–SEDDSs–30%–SOL required a reduction in process temperature in certain segments. Here, the temperature in the third segment was reduced from 140 °C to 110 °C and kept constant until the last two segments, where it was increased to 120 °C. The barrel temperature reduction was likely a response to the significant lowering of the melt viscosity when introducing SEDDSs in the process. Although SEDDSs have not yet been extensively used in HME, they likely acted as plasticizers due to their similar chemical nature as „classical“ plasticizers used in HME, such as citrate and fatty acid esters [37]. Otherwise, intrinsic polymer properties such as glass transition temperature, molecular weight, and branching as well as chemical structure may significantly impact HME process temperatures. 

### 3.3. HME–SEDDSs Characterization

#### 3.3.1. Polarized Optical Microscopy (POM)

**Figure 3:**
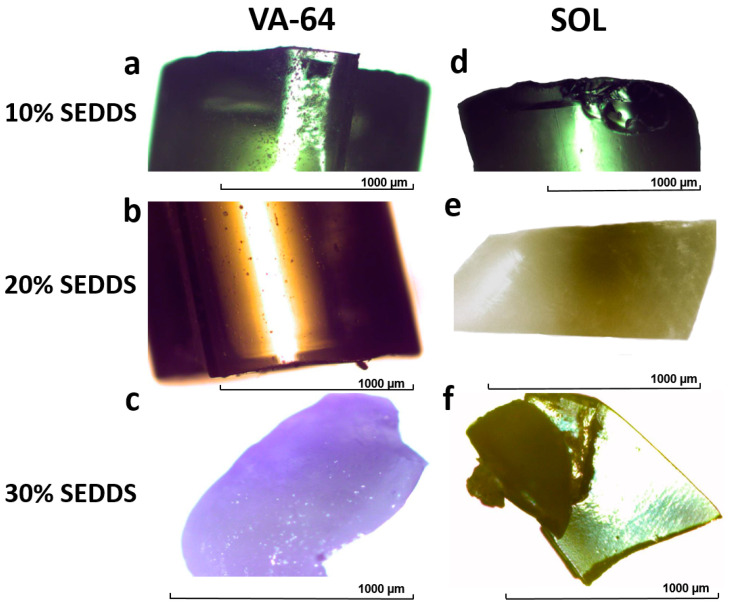
Microscope images of HME–SEDDSs: (**a**) HME–SEDDSs–10%–VA-64, (**b**) HME–SEDDSs–20%–VA-64, (**c**) HME–SEDDSs–30%–VA-64, (**d**) HME–SEDDSs–10%–SOL, (**e**) HME–SEDDSs–20%–SOL, (**f**) HME–SEDDSs–30%–SOL.

Figure 3 illustrates HME–SEDDSs prepared in 10–30% *w*/*w* SEDDSs loadings. HME–SEDDSs–10%–VA-64 resulted in a glassy, slightly turbid appearance (Figure 3a) with a similar brittle character to that of HME–SEDDSs–10%–SOL, whereas HME–SEDDSs–10%–SOL was transparent and elastic (Figure 3d). Increased SEDDSs loadings in HME–SEDDSs–20%–VA-64 resulted in the increased turbidity and a wax-like yellow–white appearance (Figure 3b), yet still a brittle nature. In contrast, HME–SEDDSs–20%–SOL appeared in a very elastic wax-like yellow–white form with a tendency to coalesce (Figure 3e). At a SEDDSs loading of 30% *w*/*w*, both polymers formed a non-uniform extrudate with an oily feel and strong tendency to coalesce. The main difference between them was that HME–SEDDSs–30%–VA-64 remained brittle and was possible to micronize with a mortar, whereas HME–SEDDSs–30%–SOL was too elastic for this purpose. 

#### 3.3.2. Thermal Phase Behavior of HME–SEDDSs Formulations 

**Figure 4:**
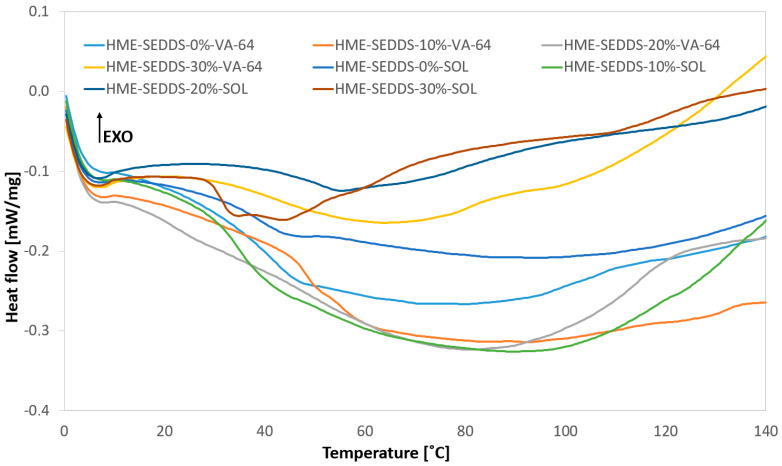
DSC thermograms of HME–SEDDSs.

The thermal properties and physical stability of the HME–SEDDSs were evaluated using mDSC. Interestingly, no endothermic peaks of Kolliphor^®^ RH40, Capmul^®^ MCM, Labrafac Lipohile^®^, and Tanscutol^®^ as well as glass transition temperature was observed in all cases (Figure 4). 

Indeed, the lack of a clear melting event, otherwise expected for crystalline SEDDS components in the solid state, suggested the formation of a homogeneous mixture of SEDDSs and polymers with no phase separation, indicating a compatibility between lipids and polymers in the used ratios. These findings are also supported by the POM images above. Interestingly, only in the case of HME–SEDDSs–30%–SOL, a broad endothermic event of a SEDDSs component was visible.

#### 3.3.3. Crystallinity of HME–SEDDSs Observed Via WAXS

**Figure 5:**
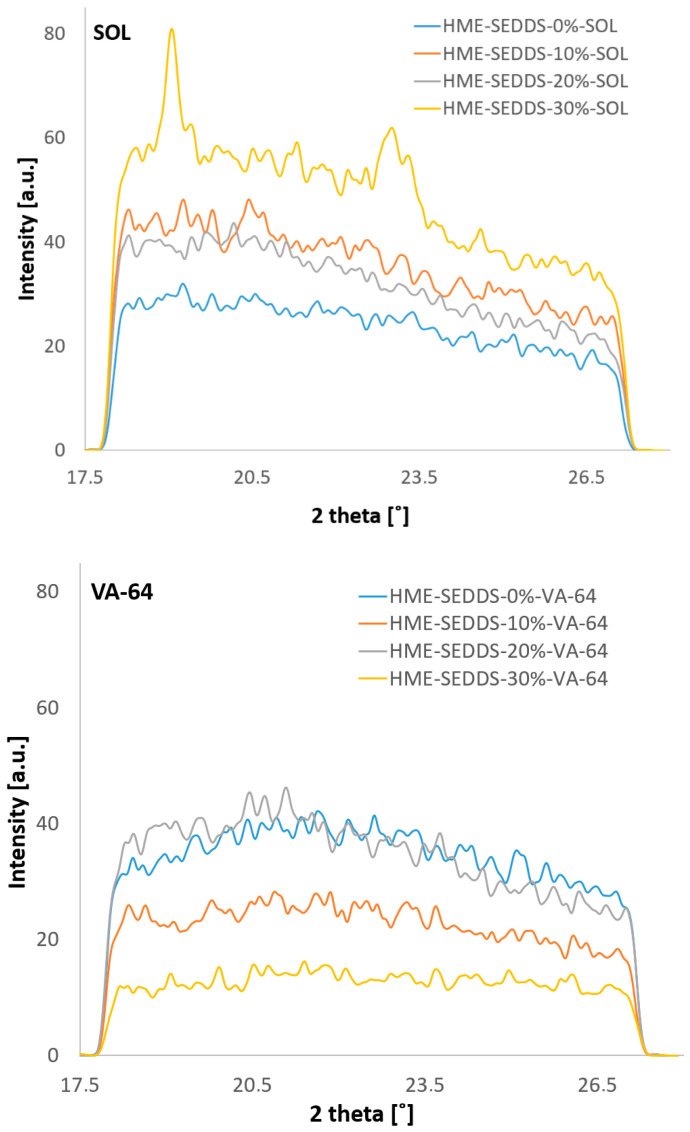
WAXS profiles of HME–SEDDSs–SOL and HME–SEDDSs–VA-64 formulations.

The WAXS results demonstrate the absence of sharp Bragg peaks in the case of HME–SEDDSs extruded with VA-64 (Figure 5). However, for HME–SEDDSs–30%–SOL, Bragg peaks at two theta values of ~19 and ~23 were observed, whereas no Bragg peaks were observed in HME–SEDDSs–10%–SOL and HME–SEDDSs–20%–SOL. This finding suggests a homogeneous phase of HME–SEDDSs–10%–SOL and HME–SEDDSs–20%–SOL. On the contrary, in HME–SEDDSs–30%–SOL, the Bragg peaks were likely due to the semi-crystalline structures of Kolliphor^®^ RH40 at room temperature. The presence of Bragg peaks suggests a possible phase separation of the SEDDSs, which could be accounted to lower SEDDSs formulation stabilization or specific micellar structure of SOL, facilitating the crystalline dispersion. In addition, the results are in line with the observation of higher emulsification time for HME–SEDDSs–SOL as well as with the more prominent endotherms in the DSC thermogram, suggesting the reduced miscibility of SOL with SEDDSs at a 70:30 *w*/*w* ratio. 

Overall, with the exception of HME–SEDDSs–30%–SOL, the WAXS and DSC results demonstrated the presence of amorphous structures in all the other formulations. Thus, the present strategy can be used for the formation of amorphous-stable structures of polymer–SEDDSs systems, with the possibility of modulating the crystalline SEDDSs component by increasing the SEDDSs in the SOL system.

#### 3.3.4. Trends in Emulsification Efficiency among HME–SEDDSs Formulations

HME-SEDDSs were evaluated for their emulsification properties. First, liquid SEDDSs were used as a control, for which a rapid self-emulsification in less than 15 s was observed. This is in accordance with the published results [7]. Moreover, differences in emulsification times in HME–SEDDSs were observed. Increasing SEDDSs from 10% to 30% *w*/*w* did not significantly affect the emulsification times in both polymers. However, the polymer matrices exhibited having a greater impact on the emulsification times of HME–SEDDSs. The HME–SEDDSs–VA-64 samples were emulsified in deionized water at 37 °C between 2 and 3 min, whereas the HME–SEDDSs–SOL samples required about 22 min to emulsify. The difference in emulsification times between HME–SEDDSs–VA-64 and HME–SEDDSs–SOL is likely related to intrinsic polymer properties and is not merely a function of the SEDDSs loading. 

#### 3.3.5. Droplet Size and Polydispersity Index (PDI) Obtained from HME–SEDDSs

Table 2 shows the droplet size of the HME–SEDDSs emulsified in 0.4% *w/v* and 2.0% *w/v* concentrations at 25 °C and 37 °C, respectively. The experimental conditions were chosen based on previous observations, where 0.4% [35] *w/v* and 2.0% SEDDSs concentrations [6,7,30,38] were tested. Deionized water was chosen as a simple dissolution medium to exclude potential interactions of HME–SEDDSs with the medium composition and potential pH impact. Moreover, most SEDDSs composed of non-ionic components and neutral zeta potential were shown to be independent of pH and dissolution medium composition [39]. 

For HME–SEDDSs–VA-64, no temperature dependency on droplet size and PDI was observed across the SEDDSs loadings. In contrast, the microemulsion properties of SOL–HME–SEDDSs changed with the temperature in HME–SEDDSs–10%–SOL and HME–SEDDSs–20%–SOL, where the mean droplet size between 60 and 70 nm at 25 °C was increased up to 140–150 nm at 37 °C. Additionally, the mean droplet size of HME–SEDDSs–30%–SOL increased with an increasing temperature from 120 nm to 146 nm. Moreover, a notable dilution effect was observed in SOL–HME–SEDDSs when comparing 0.4% *w/v* and 2.0% *w/v* samples at 37 °C. Interestingly, SOL–HME–SEDDSs, at both concentrations, showed no dilution effect at 25 °C, wherein the droplet size ranged between 60 and 70 nm. In contrast, a dilution effect was notable in HME–SEDDSs–VA-64, wherein a two-fold mean droplet size increase was observed in the 2.0% *w/v* sample compared with the 0.4% *w/v* sample. It is worth noting that, when developing SEDDSs for oral administration, the dilution effect should be considered in the formulation design. Typical liquid SEDDSs are insensitive to medium pH and dilution changes. In one study, cepharanthine SEDDSs were prepared in different dilutions at different temperatures. It was shown that the SEEDSs mean droplet size remained the same under 50, 100, and 200-fold dilutions with deionized water at 4 °C and room temperature [40]. However, since HME–SEDDSs contained higher amounts of polymers, their impact likely overweighed the effect of the SEDDSs. 

Additionally, the concentration and temperature dependency of the emulsification process and microemulsion properties may negatively influence the drug delivery system’s performance. The emulsification properties of the formulations may change under harsh and variable in vivo gastrointestinal conditions, leading to formulation instabilities. This may result in erratic drug release from the oily droplet core, exposing it to chemical degradation but also diminishing the drug solubilization and permeation potential. 

**Figure 6:**
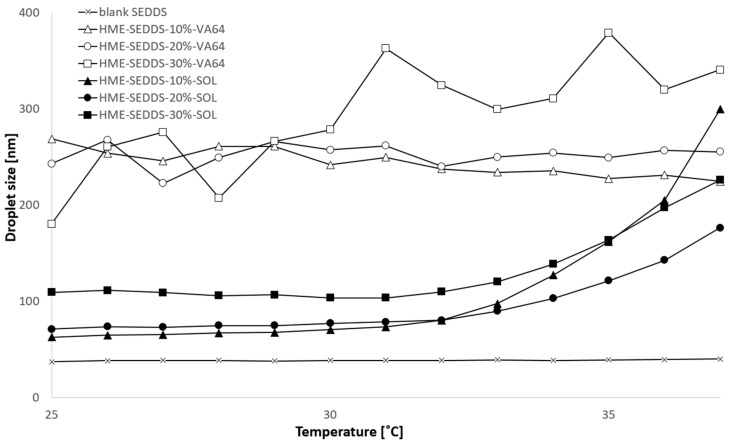
Droplet size change in 2% *w/v* emulsions of HME–SEDDSs during temperature increase from 25 °C to 37 °C.

In addition, the droplet size of the 2.0% *w/v* HME–SEDDSs was measured between 25 and 37 °C. Figure 6 shows the droplet size increase in HME–SEDDSs–SOL with increasing temperature, whereas HME–SEDDSs–VA-64 exhibited a consistent mean droplet size throughout the entire measurement range. As a control, the mean droplet size of 2% *w/v* liquid SEDDSs was determined, showing consistent droplet size and no temperature dependency. 

#### 3.3.6. Transmittance and Cloud Point 

**Figure 7:**
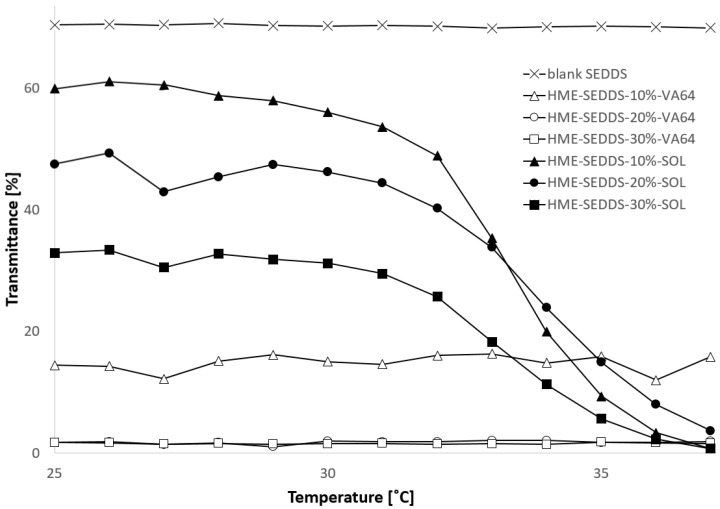
Transmittance change in 2% *w/v* emulsions of HME–SEDDSs during temperature increase from 25 °C to 37 °C. Disclaimer: HME–SEDDSs–20%–VA-64 (○) and HME–SEDDSs–30%–VA-64 (□) are overlapping.

Considering the microemulsion stability under in vivo conditions, the cloud point of the HME–SEDDSs was determined. The cloud point is defined as the temperature or temperature range wherein SEDDSs lose their self-emulsifying properties or become thermodynamically unstable, which may lead to phase separation or inversion [41]. In laboratory settings, the cloud point was visually seen as an increase in sample turbidity or reduction in its transmittance. Figure 7 shows the reduction of 2% *w/v* HME–SEDDSs transmittance with increasing medium temperature. Similar than the above regarding mean droplet size, HME–SEDDSs–VA-64 remained relatively stable with neglectable transmittance change with increasing temperature in the measured range. In contrast, HME–SEDDSs–SOL clearly showed a cloud point at about 33 °C, which was also confirmed visually, where a bluish-transparent sample turned milky-white between 32.8 and 33.6 °C. Moreover, a cloud point was observed in all the HME–SEDDSs–SOL samples, indicating that SEDDSs have no direct impact on HME–SEDDSs’ cloud point. These findings correlate with those on mean droplet size above, wherein the sample cloud point showed significant changes in the mean droplet size. This showcases that the selection of appropriate polymers could be considered as a critical quality attribute in developing HME–SEDDSs. 

## 4. Conclusions

**Figure 8:**
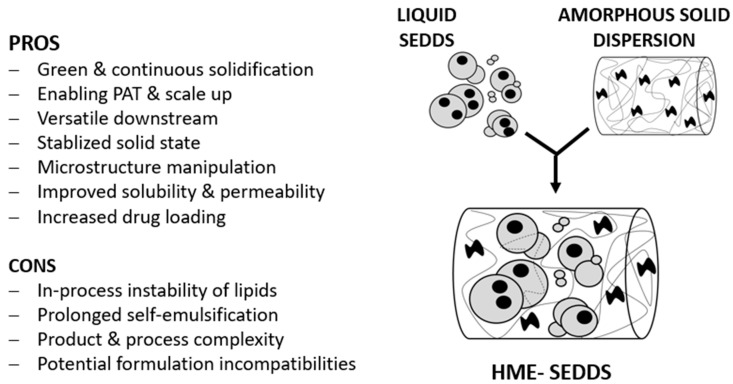
Pros and cons of the prepared HME–SEDDSs compared with conventional liquid SEDDSs and amorphous solid dispersions.

This study showed for the first time that a continuous solidification of SEDDSs via HME using exclusively a polymer matrix was feasible in the pilot scale using a split feeding approach. VA-64 and SOL were chosen as typical HME polymers with suitable SEDDSs-binding properties. Different SEDDSs loadings of 10, 20, and 30% *w*/*w* were achieved under chosen HME settings, wherein processing temperatures between 110 and 160 °C were applied. A plasticizing effect was observed with increasing SEDDSs amounts. The prepared HME–SEDDSs, with the exception of HME–SEDDSs–30%–SOL, were amorphous and exhibited a homogenous structure, indicating a compatibility between lipids and polymers. In addition, the emulsification properties in different dilutions and temperature ranges were likely dominated by the polymer, wherein in HME–SEDDSs–SOL a clear mean droplet size change when passing its cloud point of ~33 °C was seen. In contrast, HME–SEDDSs–VA-64 were not affected by dissolution medium temperature. In conclusion, HME seems to be a valid continuous technique for the solidification of SEDDSs. Figure 8 shows the pros and cons of the prepared HME–SEDDSs compared with the conventional liquid SEDDSs and amorphous solid dispersions. 

## Figures and Tables

**Table 1:** Composition of liquid SEDDSs and HME–SEDDSs prepared in this study.

Formulation	Composition
Liquid SEDDSs	30% Labrafac^®^ lipophile 30% Kolliphor^®^ RH40 30% Capmul^®^ MCM 10% Transcutol^®^
HME–SEDDSs 10% BLANK	90% Polymer * 3% Labrafac^®^ lipophile 3% Kolliphor^®^ RH40 3% Capmul^®^ MCM 1% Transcutol^®^
HME–SEDDSs 20% BLANK	80% Polymer * 6% Labrafac^®^ lipophile 6% Kolliphor^®^ RH40 6% Capmul^®^ MCM 2% Transcutol^®^
HME–SEDDSs 30% BLANK	70% Polymer * 9% Labrafac^®^ lipophile 9% Kolliphor^®^ RH40 9% Capmul^®^ MCM 1% Transcutol^®^

* Kollidon^®^ VA 64 and Soluplus^®^ were used as polymers.

**Table 2:** Mean droplet size and polydispersity index of liquid SEDDSs and HME–SEDDSs after 0.4% and 2.0% w/V dilution in deionized water.

Concentration	0.4% *w/v*				2.0% *w/v*			
Temperature	25 °C		37 °C		25 °C		37 °C	
	Droplet Size (nm)	PDI	Droplet Size (nm)	PDI	Droplet Size (nm)	PDI	Droplet Size (nm)	PDI
Blank SEDDSs	31.8	0.051	35.8	0.035	32.5	0.048	36.0	0.041
HME–SEDDSs–10%–VA-64	122.7	0.214	136.1	0.197	257.1	0.255	286.0	0.256
HME–SEDDSs–20%–VA-64	152.9	0.193	170.6	0.132	259.1	0.246	234.4	0.269
HME–SEDDSs–30%–VA-64	115.7	0.251	129.2	0.244	226.3	0.279	285.8	0.271
HME–SEDDSs–10%–SOL	61.8	0.061	90.2	0.089	63.9	0.047	139.1	0.152
HME–SEDDSs–20%–SOL	68.7	0.100	95.8	0.085	71.7	0.145	150.4	0.194
HME–SEDDSs–30%–SOL	73.5	0.128	95.1	0.119	120.1	0.264	145.8	0.182

## Abbreviations

ASD	Amorphous solid dispersion
DSC	Differential scanning calorimetry
GMP	Good manufacturing practice
HME	Hot melt extrusion
HME–SEDDS	Solid SEDDSs prepared by hot melt extrusion
HPC	Hydroxy propyl cellulose
HPMCAS	Hydroxy propyl methylcellulose-acetate/succinate
LOD	Loss on drying
MCC	Microcrystalline cellulose
PAT	Process analytical technology
PDI	Polydispersity index
PL_630	Plasdone^®^ 630
PL_630_U	Plasdone^®^ 630 Ultra
SEDDSs	Self-emulsifying drug delivery systems
SEDEXSOL	Self-emulsifying delivery via extrusionSoluplus^®^
SOL-HME–SEDDSs	Solid SEDDSs prepared by hot melt extrusion with Soluplus^®^
VA-64	Kollidon^®^ VA-64, copovidone
VA-64-HME–SEDDSs	Solid SEDDSs prepared by hot melt extrusion with Kollidon VA-64^®^
WAXS	Wide-angle X-ray scattering
ZSK18	Co-rotating closely intermeshing twin screw extruder from Coperion with a nominal screw diameter of 18 mm
